# A phase 2 study of isatuximab monotherapy in patients with multiple myeloma who are refractory to daratumumab

**DOI:** 10.1038/s41408-021-00478-4

**Published:** 2021-05-12

**Authors:** Joseph Mikhael, Karim Belhadj-Merzoug, Cyrille Hulin, Laure Vincent, Philippe Moreau, Cristina Gasparetto, Ludek Pour, Ivan Spicka, Ravi Vij, Jeffrey Zonder, Djordje Atanackovic, Nashat Gabrail, Thomas G. Martin, Aurore Perrot, Samira Bensfia, Qilong Weng, Claire Brillac, Dorothée Semiond, Sandrine Macé, Kathryn P. Corzo, Xavier Leleu

**Affiliations:** 1 Translational Genomics Research Institute, City of Hope Cancer Center, Phoenix, AZ USA; 2 Unité Hémopathies Lymphoïdes, CHU Henri Mondor, Créteil, France; 3 Service d’hématologie, CHRU Hôpitaux de Brabois, Nancy, France; 4 Département d’hématologie Clinique, Centre Hospitalier Universitaire de Montpellier, Montpellier, France; 5 Hematology Department, Nantes University Hospital, Nantes, France; 6 Duke Cancer Institute, Duke University, Durham, NC USA; 7 Department of Internal Medicine, Hematology and Oncology, University Hospital Brno, Brno, Czech Republic; 8 1st Department of Medicine—Department of Hematology First Faculty of Medicine Charles University and General Hospital in Prague, Prague, Czech Republic; 9 Division of Medical Oncology, Washington University, St Louis, MO USA; 10 Department of Oncology, Karmanos Cancer Institute, Wayne State University, Detroit, MI USA; 11 Department of Medicine, Bone Marrow Transplant, University of Maryland Greenebaum Cancer Center, Baltimore, MD USA; 12 Gabrail Cancer Center, Canton, OH USA; 13 Helen Diller Family Comprehensive Cancer Center, University of California San Francisco, San Francisco, CA USA; 14 CHU de Toulouse, IUCT-O, Université de Toulouse, UPS, Service d’hématologie, Toulouse, France; 15 Sanofi Global Oncology, Cambridge, MA USA; 16 Sanofi Clinical Sciences and Operations, Beijing, China; 17 Sanofi Translational Medicine and Early Development, Paris, France; 18 CHU and CIC Inserm1402, Poitiers, France; 19 Present Address: Takeda Pharmaceuticals, Cambridge, MA USA

**Keywords:** Myeloma, Drug development

Dear Editor,

The use of novel therapies, such as immunomodulatory drugs (IMiDs), proteasome inhibitors (PIs), and monoclonal antibodies (mAbs; ie, isatuximab, daratumumab, elotuzumab), has significantly improved the outcomes of multiple myeloma (MM) patients. However, MM remains largely incurable, with the majority of patients becoming refractory to available therapies and eventually relapsing.

Isatuximab binds to a specific epitope on CD38 and selectively induces MM cell death through several mechanisms, including antibody-dependent cell-mediated cytotoxicity, antibody-dependent cellular phagocytosis, and complement-dependent cytotoxicity^1^. Isatuximab is the only anti-CD38 mAb that induces direct apoptosis in MM cell lines in the absence of cross-linking agents and independently of effector cells^2,3^. Additionally, isatuximab inhibits CD38 enzymatic activity more effectively than daratumumab^3^, resulting in decreased adenosine production, and may alleviate the immunosuppressive microenvironment of the bone marrow niche in MM patients^4^. Isatuximab also induces indirect antitumor activity through the elimination of CD38^+^ immunosuppressive regulatory T cells and through an “in vivo vaccination” effect (reviewed by Martin et al.^4^). Isatuximab is approved in combination with pomalidomide and dexamethasone (Pd) in the USA, Europe, and Asia for the treatment of adult patients with relapsed/refractory MM (RRMM) who have received at least two prior therapies, including lenalidomide and a PI^5–7^. Daratumumab is a different anti-CD38 mAb and is approved for use in MM as monotherapy and in combination regimens.

This Phase 1/2 study (ClinicalTrial.gov identifier, NCT02514668) was conducted in 19 sites in the USA and Europe. Patients were treated with isatuximab 20 mg/kg every week for 4 weeks and every other week thereafter. The safety and efficacy of isatuximab in Part A (Phase 1) were generally comparable to other isatuximab studies in MM^8,9^. This report presents Part B (Phase 2), assessing the response, safety, pharmacokinetics, and immunogenicity of isatuximab in daratumumab-refractory RRMM patients.

The objective of Part B was to assess the clinical benefit of isatuximab monotherapy in daratumumab-refractory RRMM patients, as measured by overall response rate (ORR). To our knowledge, this was the first prospective study evaluating the ability of anti-CD38 mAb monotherapy to overcome the refractoriness of of patients to a different anti-CD38 mAb.

The study design is summarized in Supplementary Fig. S1. Eligible patients had RRMM and progressed on/after standard therapy, including an IMiD and a PI, and had (1) ≥3 prior cycles of daratumumab treatment with ≥6 weeks from the last daratumumab treatment to the first study treatment or (2) ≥2 cycles of daratumumab treatment if another therapy was given between daratumumab and isatuximab, with ≥12 weeks from the last daratumumab treatment to the first study treatment.

Patient baseline characteristics (*N* = 32) are shown in Table 1. Overall, 75% of patients were ≥65 years old and 34.4% of patients had Eastern Cooperative Oncology Group stage II or III. One-third (31.3%) had International Staging System stage III at study entry. High-risk cytogenetic status was determined for 13/32 patients based on ≥1 del17p (3/13 [23.1%] patients) or t(4;14) (3/13 [23.1%] patients) or t(14;16) (0 patients). Patients were very heavily pretreated, with a median of 7 (range, 2–14) prior lines; two-thirds of patients (68.8%) received ≥5 prior lines of therapy. All patients were refractory (showed progression of disease on treatment or within 60 days of treatment end date) to daratumumab alone or in combination and to their last treatment line. Overall, 75% of patients were double refractory and 28% were quad- or penta-refractory. Over 50% previously received daratumumab in combination with other therapies. The majority of patients (60%) received daratumumab combination therapy just prior to isatuximab treatment, 62.5% had <6 months between last daratumumab and first isatuximab dose, and 15% received ≥2 prior daratumumab lines.

**Table 1:** Baseline patient characteristics at study entry in the all-treated population.

	Isatuximab 20 mg/kg QW/Q2W (*N* = 32)
Age (years)	
Median (range)	70.5 (51–84)
<65 years, *n* (%)	8 (25.0)
65–74 years, *n* (%)	14 (43.8)
≥75 years, *n* (%)	10 (31.3)
Median time from diagnosis to first dose, years (range)	7.1 (1.2–19.4)
MM subtype, *n* (%)	
IgG	13 (40.6)
IgA	8 (25.0)
IgM	0 (0)
Kappa light chain only	6 (18.8)
Lambda light chain only	5 (15.6)
ISS stage^a^, *n* (%)	
Stage I	12 (37.5)
Stage II	9 (28.1)
Stage III	10 (31.3)
Unknown	1 (3.1)
ECOG performance status, *n* (%)	
0	5 (15.6)
1	16 (50.0)
2	10 (31.3)
3	1 (3.1)
Cytogenetic risk^b^, *n* (%)	
High-risk CA	5 (15.6)
Standard-risk CA	8 (25.0)
Unknown or missing	19 (59.4)
Number of prior lines of therapy	
Median (range)	7.0 (2–14)
Number of prior lines by patient by category, *n* (%)	
<5	10 (31.3)
≥5	22 (68.8)
Prior therapy, *n* (%)	
Alkylating agent	31 (96.9)
IMiD agent	31 (96.9)
PI agent	32 (100)
PI and IMiD agent	31 (96.9)
Dara	32 (100)
Refractory status, *n* (%)	
IMiD refractory	29 (90.6)
PI refractory	26 (81.3)
PI and IMiD refractory	24 (75.0)
Quad-refractory (RPVK)	9 (28.1)
Penta-refractory (RPVK–Dara)	9 (28.1)
Refractory to last line	32 (100)
Refractory to Dara	32 (100)
Number of Dara lines, *n* (%)	
1	27 (84.4)
2	4 (12.5)
3	1 (3.1)
Dara therapy type, *n* (%)	
Monotherapy	15 (46.9)
Combination therapy	17 (53.1)
Duration of Dara treatment by category (months), *n* (%)	
<6 months	14 (43.8)
≥6 months	18 (56.3)
Best response with Dara, *n* (%)	
Complete response	3 (9.4)
Very good partial response	7 (21.9)
Partial response	10 (31.3)
Minimal response	2 (6.3)
Stable disease	6 (18.8)
Progressive disease	4 (12.5)
Median time from last dose Dara to first Isa, weeks (range)	13.07 (6–80.7)
<12 weeks	14 (43.8)
≥12 weeks	18 (56.3)
<24 weeks	20 (62.5)
≥24 weeks	12 (37.5)
<48 weeks	27 (84.4)
≥48 weeks	5 (15.6)
Dara as last line prior to Isa, *n* (%)	19 (59.4)

*CA* chromosomal abnormalities, *COPD* chronic obstructive pulmonary disease, *d* dexamethasone, *Dara* daratumumab, *ECOG* Eastern Cooperative Oncology Group, *Ig* immunoglobulin, *IMiD* immunomodulatory drug, *Isa* isatuximab, *ISS* International Staging System, *K* carfilzomib, *MM* multiple myeloma, *P* pomalidomide, *PI* proteasome inhibitor, *QW/Q2W* once weekly for 4 weeks, then every other week, *R* lenalidomide, *V* bortezomib.

^a^ISS staging was derived based on the combination of serum β2-microglobulin and albumin.

^b^High-risk CA was defined as the presence of del(17p), and/or t(4;14), and/or t(14;16) by fluorescence in situ hybridization. Cytogenetic analysis was performed by a central laboratory with a cut-off of 10% of analyzed plasma cells for del(17p), and 15% of analyzed plasma cells for t(4;14) and t(14;16).

The median duration of exposure to isatuximab was 8.3 weeks (range, 1–74 weeks; Supplementary Table S1). Eleven patients received dexamethasone after either the second or the fourth cycle, depending on the observed response. Two of the 32 enrolled patients were not evaluable for response. Objective ORR was not reached (Supplementary Table S2). One (3.1%) patient had minimal response (MR) and 17 (53.1%) patients had stable disease (SD). The disease control rate (DCR, defined as ≥MR or SD ≥8 weeks) was 37.5%. A long duration of treatment and prolonged SD were observed in some patients (Fig. 1A). One patient had SD and a treatment duration of 74 weeks (18.5 months), whereas three patients had a treatment duration of ≥6 months and three patients of ≥3 months.

**Fig. 1:**
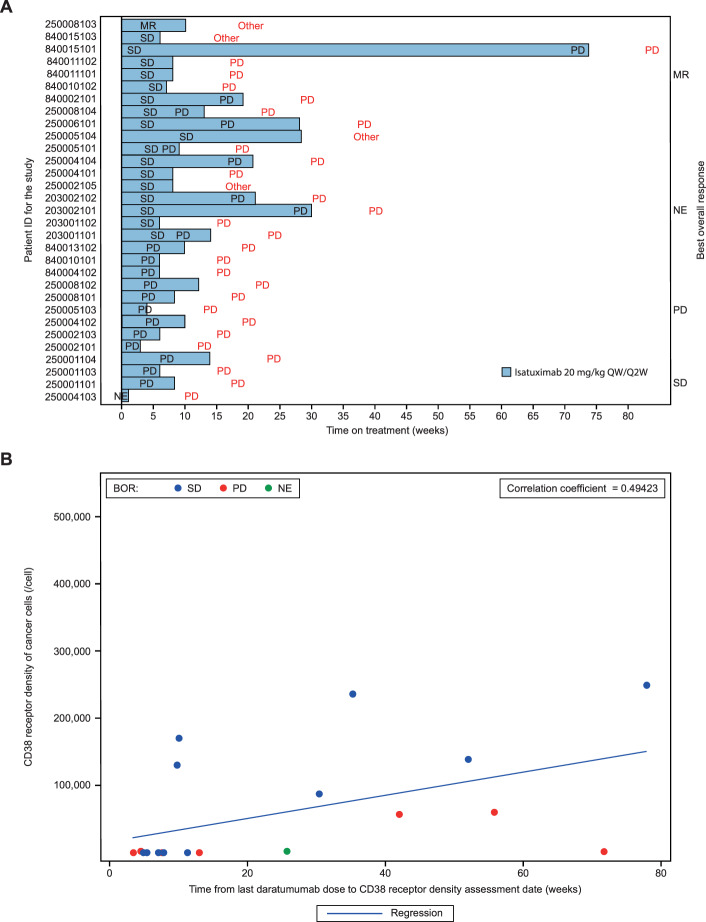
Isatuximab monotherapy treatment response correlation with CD38 receptor density. **A** Swimmer plot of time on treatment with isatuximab monotherapy. Each bar represents one of the 31 patients evaluable for response in the study (i.e., patients who completed at least one cycle of treatment and who had at least one disease assessment or patients with clinical progression or patients who died within 30 days of first dose due to disease progression). One out of the 31 patients had no evaluable response. Text in red font corresponds to the reason for treatment discontinuation. **B** Higher baseline CD38 receptor density was associated with longer periods from the last daratumumab dose to the CD38 receptor density assessment date. The scatter plot shows the CD38 receptor density data and time from last daratumumab dose to CD38 receptor density assessment date (all-treated population). The CD38 receptor density of cancer cells was measured at baseline by quantitative flow cytometry in bone marrow aspirate from 19 of 32 patients. The estimated CD38 receptor density reflects the number of free receptors per cell accessible for isatuximab binding and not the total CD38 receptor density. *BOR* best overall response, *MR* minimal response, *NE* not evaluable, *PD* progressive disease, *QW/Q2W* once weekly for 4 weeks, then every other week, *SD* stable disease.

Importantly, the DCR doubled in patients with the longest interval between the last daratumumab dose and the first isatuximab dose; 58.3% with a washout ≥6 months vs 28.6% with a washout <3 months (Supplementary Table S3). DCR was high (72.7%) among the 11 patients who received dexamethasone with isatuximab.

Median progression-free survival was 1.6 months (95% CI: 1–3.2) and median overall survival was 10.7 months (95% CI: 8–19, Supplementary Table S2).

Isatuximab and daratumumab pharmacokinetics analyses are described in the Supplementary Appendix (Fig. S2).

Primary resistance to daratumumab or isatuximab has been linked to CD38 receptor density (RD) and there is a trend toward higher response rates with increasing CD38 RD (reviewed by Martin et al.^4^). Therefore, we measured the CD38 RD using flow cytometry with an antibody competing with daratumumab (the estimated CD38 RD reflects the number of free receptors per cell accessible for isatuximab binding). Higher baseline CD38 RD was associated with longer periods from the last daratumumab dose to the CD38 RD assessment date (Fig. 1B). However, these data should be interpreted with caution, as the test did not permit accurate measurement of CD38 RD. Collectively, CD38 RD values from 0 to <5000 RD/cell were detected in 11 patients who had <20 weeks from last daratumumab dose to the CD38 RD assessment date. This may have been due to shedding, aggregation, internalization, or 100% occupation of CD38 receptors on bone marrow cells by daratumumab (Fig. 1B). The three patients with a CD38 RD of ≥150,000/cell had a better DCR of 66.7% compared with 37.5% for the 16 patients with a CD38 RD of <150,000/cell. This threshold corresponds to the median value reported in the isatuximab monotherapy study^8^.

The safety profile in this study is described in the Supplementary Appendix and was similar to that reported in prior isatuximab monotherapy studies^8,9^, with no new safety concerns (Supplementary Tables S4 and S5). A total of 28 (87.5%) and 16 (50.0%) patients had treatment-emergent adverse events (TEAEs) and a Grade ≥3 TEAE, respectively. There were very few interrupted infusions (5/257). Infusion reactions (IR, all Grades 1–2) were reported in 18.8% of patients. Such lower IR incidence compared with other isatuximab studies is presumably due to prior exposure to another anti-CD38 mAb.

This isatuximab monotherapy study included very heavily pretreated and daratumumab-refractory RRMM patients, with the majority treated with daratumumab combination therapy and as last line. Based on its recent approvals, isatuximab will predominantly be used in clinical practice as combination therapy and in earlier lines, and results of ongoing clinical trials may shed light on the effectiveness of isatuximab combination therapy in daratumumab-refractory RRMM patients. The Phase 1b Part B study (ClinicalTrials.gov, NCT02283775) of isatuximab combined with Pd administered by a fixed-volume infusion method enrolled 7 (of 47) RRMM patients with prior daratumumab exposure. All seven patients were daratumumab-refractory and none of the patients received daratumumab as last regimen. At interim data analysis, six of those seven patients were evaluable for response and one had partial response, two had MR, and three had SD^10^. A real-world analysis of RRMM patients with prior daratumumab therapy demonstrated that 10/15 patients experienced a response of MR or better with isatuximab in combination with Pd treatment^11^. In line with these observations, a retrospective study showed that patients refractory to daratumumab and pomalidomide exhibited an ORR of 33% when retreated with daratumumab combined with Pd, indicating that the combination of IMiDs may overcome anti-CD38 mAb refractoriness by increasing plasma cell CD38 expression and enhancing T-cell and NK-cell responses^12^.

In conclusion, this cohort of daratumumab-refractory RRMM patients treated with isatuximab monotherapy was heavily pretreated, with a median of 7 (range, 2–14) prior lines and 100% were refractory to daratumumab. The majority was recently exposed to daratumumab combination therapy, with ~60% having the last daratumumab dose within 6 months and as the last line of therapy. Although there were no objective responses, 1 (3.1%) patient achieved MR and 17 (53.1%) patients had SD as best overall response, with the longest duration of SD being 18.5 months. The DCR in this heavily pretreated population, refractory to last line and to daratumumab, was 37.5%. Better responses were observed in patients with longer intervals (in particular, ≥6 months) from the last daratumumab dose to the first isatuximab dose, as measured by a higher DCR (26.4% [last dose <6 months] vs 58.3% [last dose ≥6 months] vs 60.0% [last dose ≥12 months]). Further study regarding the use of isatuximab post daratumumab, or vice versa, is required to better understand the optimal timing and sequencing of CD38 mAbs in MM.

## Supplementary information

Supplemental Appendix
