# Supplementary material for: A phase 2 study of isatuximab monotherapy in patients with multiple myeloma who are refractory to daratumumab
Source: Blood Cancer J. 2021 May 12;11(5):89. doi: 10.1038/s41408-021-00478-4 (PMC8116334; doi:10.1038/s41408-021-00478-4)
Supplement: Supplementary file 1 — Supplemental Appendix [file 41408_2021_478_MOESM1_ESM.docx]

**Supplementary Information**

**A phase 2 study of isatuximab monotherapy in patients with multiple myeloma who are refractory to daratumumab**

Joseph Mikhael,^1^ Karim Belhadj-Merzoug,^2^ Cyrille Hulin,^3^ Laure Vincent,^4^ Philippe Moreau,^5^ Cristina Gasparetto,^6^ Ludek Pour,^7^ Ivan Spicka,^8^ Ravi Vij,^9^ Jeffrey Zonder,^10^ Djordje Atanackovic,^11^ Nashat Gabrail,^12^ Thomas G. Martin,^13^ Aurore Perrot,^14^ Samira Bensfia,^15^ Qilong Weng,^16^ Claire Brillac,^17^ Dorothée Semiond,^17^ Sandrine Macé,^17^ Kathryn P. Corzo,^18*^ Xavier Leleu^19^

^1^Translational Genomics Research Institute, City of Hope Cancer Center, Phoenix, AZ, USA

^2^Unité Hémopathies Lymphoïdes, CHU Henri Mondor, Créteil, France

^3^Service d’hématologie, CHRU Hopitaux de Brabois, Nancy Cedex, France

^4^Département d'hématologie clinique, Centre Hospitalier Universitaire de Montpellier, Montpellier, France

^5^Hematology Department, Nantes University Hospital, Nantes, France

^6^Duke Cancer Institute, Duke University, Durham, NC, USA

^7^Department of Internal Medicine, Hematology and Oncology, University Hospital Brno, Brno, Czech Republic

^8^1st Department of Medicine – Department of Hematology First Faculty of Medicine Charles University and General Hospital in Prague, Prague, Czech Republic

^9^Division of Medical Oncology, Washington University, St Louis, MO, USA

^10^Department of Oncology, Karmanos Cancer Institute, Wayne State University, Detroit, MI, USA

^11^Department of Medicine, Bone Marrow Transplant, University of Maryland Greenebaum Cancer Center, Baltimore, MD, USA

^12^Gabrail Cancer Center, Canton, OH, USA

^13^Helen Diller Family Comprehensive Cancer Center, University of California San Francisco, San Francisco, CA, USA

^14^CHU de Toulouse, IUCT-O, Université de Toulouse, UPS, Service d’hématologie, Toulouse, France

^15^Sanofi Global Oncology, Cambridge, MA

^16^Sanofi Clinical Sciences & Operations, Beijing, China

^17^Sanofi Translational Medicine & Early Development, Paris, France

^18^Sanofi (at the time of the study)

^19^CHU and CIC Inserm 1402, Poitiers, France

* Affiliation at time of study, currently Takeda Pharmaceuticals, Cambridge, MA, USA

**Correspondence:**

Xavier Leleu, MD, PhD

Service d'hématologie et thérapie cellulaire, Hôpital de la Milétrie, Faculté de médecine
and
Inserm CIC 1402, CHU

2 rue de la Milétrie, 86021 Poitiers Cedex, France

Tel: +33 549 443 717

Fax: +33 549 443 863

E-mail: Xavier.LELEU@chu-poitiers.fr

**Methods**

Determination of sample size

The sample size calculation followed Simon's 2-stage MinMax design. A sample of 40 patients evaluable for treatment response provided 90% power to reject the null hypothesis that the true ORR was ≤10% if the true ORR was ≥25%, based on a 1-sided exact binomial test at a significance level of 0.1. The first 27 treated patients who were evaluable for ORR were to be included in Stage 1 analysis. If ≤2 responses were observed, consideration was given to stop accrual. If ≥3 responses were observed, the study was to proceed to Stage 2. In Stage 2, 13 additional patients were to be enrolled. If at least 7 responses were observed among the 40 treated and evaluable patients, the null hypothesis was to be rejected.

Study design and participants

This was a multicenter, open-label, nonrandomized, phase 2 study conducted in 19 sites in the USA, France, Czech Republic, and Estonia. The study comprised of two parts, of which Part A was the dose-escalation phase and Part B was the expansion cohort. The protocol was approved by institutional review boards and independent ethics committees of all participating institutions.

Eligible patients were at least 18 years old with relapsed/refractory multiple myeloma (RRMM), had progressed on/after standard therapy, including an immunomodulatory drug and a proteasome inhibitor, and had received (i) at least 3 prior cycles of daratumumab treatment with at least 6 weeks from the last treatment with daratumumab to the first study treatment or (ii) at least 2 cycles of daratumumab treatment in case another therapy was given between daratumumab and isatuximab, with at least 12 weeks from the last treatment with daratumumab to the first study treatment. Patients were to have measurable disease based on International Myeloma Working Group (IMWG) criteria: (i) serum M-protein ≥1 g/dL (≥0.5 g/dL if immunoglobulin [Ig] A subtype), or urine M-protein ≥200 mg/24 hours; or, (ii) in the absence of measurable M-protein, serum immunoglobulin free light chain ≥10 mg/dL, and abnormal serum immunoglobulin kappa lambda free light chain ratio. Patients must have achieved a minimal response or better to at least 1 prior line of therapy.

Patients in this study received isatuximab monotherapy intravenously at 20 mg/kg every week for 4 weeks and every other week thereafter. In selected cases, for patients who had progressive disease after 2 cycles of treatment or stable disease for 4 cycles, dexamethasone 40 mg (or 20 mg for patients ≥75 years) weekly was added upon investigator decision.

All patients received premedication with methylprednisolone 100 mg IV or orally, diphenhydramine 25–50 mg IV (or equivalent), ranitidine 50 mg IV (or equivalent) and acetaminophen 650–1000 mg orally, at least 15, but no longer than 60 minutes, prior to the start of the isatuximab infusion. Dexamethasone was used both as premedication (in replacement of methylprednisolone) and as part of study treatment.

Outcomes

The primary efficacy endpoint was overall response rate according to the IMWG uniform response criteria (defined as the proportion of patients with stringent complete response, complete response, very good partial response, and partial response) based on the investigator’s assessment. Response evaluations were performed on a monthly basis and were confirmed on 2 consecutive disease assessments.

Key secondary endpoints included duration of response, progression-free survival (PFS), disease control rate (DCR), overall survival (OS), pharmacokinetics of daratumumab (at baseline) and isatuximab (at baseline and during treatment), and CD38 receptor density as an exploratory objective.

Pharmacokinetic and CD38 receptor density measurements

Daratumumab plasma concentrations were determined using a validated enzyme-linked immunosorbent assay (ELISA) method, with a lower limit of quantification (LLOQ) of 0.5 μg/mL and an upper limit of quantitation (ULOQ) of 48 μg/mL. To measure plasma concentrations of isatuximab, a validated ELISA method was used with a LLOQ of 0.5 ng/mL. Data from Part B were pooled with data from Part A to enable a reliable estimation of the population pharmacokinetics parameters. The population pharmacokinetics analysis was performed using the stochastic approximation version of the expectation–maximization algorithm (SAEM algorithm) for nonlinear mixed-effects models implemented in MONOLIX software (2018 R1 version 5.0.0). The pharmacokinetics of isatuximab were described by a 2-compartment structural kinetic model with linear elimination from the central compartment with a combined error model, with interindividual variability and Part B versus Part A set as a covariate for all pharmacokinetics parameters.

CD38 receptor density in bone marrow samples were analyzed by quantitative flow cytometry.

Statistical analyses

The statistical evaluation for all analyses was descriptive and included patients who gave informed consent and received at least one dose (even incomplete) of isatuximab treatment. PFS (time from the date of first study treatment administration to the date of first documentation of confirmed progressive disease, symptomatic deterioration, or death), DCR (defined as ≥minimal response or stable disease ≥8 weeks), and OS (time from the date of first study treatment administration to the date of death) were analyzed by the Kaplan–Meier method.

Data sharing

Qualified researchers can request access to patient-level data and related study documents including the clinical study report, study protocol with any amendments, blank case report forms, statistical analysis plan, and dataset specifications. Patient-level data will be anonymized, and study documents will be redacted to protect the privacy of trial participants. Further details on Sanofi’s data-sharing criteria, eligible studies, and process for requesting access are at: https://www.clinicalstudydatarequest.com.

**Results**

Daratumumab pharmacokinetics analyses

Daratumumab plasma concentrations measured at the start of isatuximab treatment were quantifiable (LLOQ = 0.05 µg/mL) in 14 patients, and were measurable up to 29 weeks after the last daratumumab dose. The shorter the time between the end of daratumumab treatment and the start of isatuximab treatment, the higher the daratumumab concentrations (Supplementary Fig. S2A).

Isatuximab pharmacokinetics analyses

Isatuximab early exposure (area under the curve [AUC]) calculated over the first 4-week administration (Cycle 1, [AUC_0W_4W]) in daratumumab-refractory patients enrolled in Part B of the study was compared with daratumumab-naïve patients treated at the same dose in Part A of the study (*n*=14). A moderate increase in isatuximab exposure was observed in daratumumab-refractory patients compared with daratumumab-naïve patients, with geometric mean ratios (Part B versus Part A) around 1.33 for C_trough_ (plasma concentration before dosing) and 1.75 for AUCs.

Isatuximab early exposure (AUC_0W_4W) increased with the presence of residual daratumumab concentration (Supplementary Fig. S2B) and with a shorter time interval (≤6 months) between the last dose of daratumumab and the first dose of isatuximab (Supplementary Fig. S2C). However, the higher exposure to isatuximab for daratumumab-refractory patients was gradually reduced over the course of treatment (Cycles 1, 2, and 3) and became comparable at steady-state (Cycle 6), with median ratios (Part B vs Part A) close to 1 (Supplementary Fig. S2D). The prior daratumumab treatment effect on the isatuximab exposure seemed to be reversible through the treatment cycles.

The relative transitory higher exposure over Cycle 1 observed in patients previously treated with daratumumab could be due to less accessibility to the CD38 receptor for isatuximab (e.g., CD38 receptors on bone marrow cells still occupied by daratumumab, shedding, aggregation, and/or internalization of CD38 receptors on bone marrow cells following daratumumab treatment)^1,2^.

Safety

Overall, 28 (87.5%) patients had treatment-emergent adverse events (TEAEs) and 16 (50.0%) patients had a Grade ≥3 TEAE (Supplementary Table S4). Collectively, 13 (40.6%) patients had ≥1 serious TEAE, including 1 patient with a serious drug-related TEAE. Four patients died (1 due to sepsis, 2 progressive disease, and 1 unknown cause); however, no deaths were drug related. The most common TEAEs (all grades) reported in >10% of the patients included asthenia (21.9%), bronchitis (18.8%), and infusion reactions ([IR] 18.8%). The lower IR incidence compared with other isatuximab studies is presumably due to prior exposure to another anti‑CD38 mAb. Of the 6 patients with IR, 4 had their last daratumumab dose >10 months before the first isatuximab dose. Neither Grade ≥3 IR nor delayed IR were reported. Grade ≥3 TEAEs reported in >5% of patients consisted of bone pain, dyspnea, and cellulitis (Supplementary Table S4). Hematological laboratory abnormalities included anemia (90.6%), thrombocytopenia (71.9%), lymphopenia (68.8%), and neutropenia (40.6%; Supplementary Table S5). No febrile neutropenia or neutropenic infection were reported.

**References**

1. van de Donk, N. W. et al. CD38 antibodies in multiple myeloma: Mechanisms of action and modes of resistance. *Front. Immunol.* **9,** 2134 (2018).

2. Saltarella, I. et al. Mechanisms of resistance to anti-CD38 daratumumab in multiple myeloma. *Cells* **9,** (2020).

**Supplementary Table S1. Isatuximab exposure and dose modifications.**

|  | **Isatuximab 20 mg/kg QW/Q2W**  **(*N*=32)** |
| --- | --- |
| Median number of cycles, *n* (range) | 2 (1–18) |
| At least 4 cycles, *n* (%) | 8 (25) |
| Median duration of exposure, weeks (range) | 8.3 (1–74) |
| Median duration of follow-up, months (range) | 1.9 (0.8–17) |
| OS Median duration of follow-up, months (range) | 4.7 (0.4–18.5) |
| Patients with at least 1 cycle delay, *n* (%) | 9 (28.1) |
| Patients with at least 1 dose omission, *n* (%) | 1 (3.1) |
| Patients with at least 1 infusion interrupted, *n* (%) | 5 (15.6) |
| Median time from infusion start to first interruption, min (range) | 91 (50–105) |
| Duration of infusion | |
| Median first infusion, hours  Median subsequent infusions, hours | 5  4.5 |

*OS* overall survival, *QW/Q2W* once weekly for 4 weeks, once every other week thereafter.

**Supplementary Table S2. Efficacy of isatuximab monotherapy after daratumumab treatment.**

|  | **Isatuximab 20 mg/kg QW/Q2W**  **(*N*=32)** | **Addition of dexamethasone**  **(*n*=11)** | |
| --- | --- | --- | --- |
| **Best overall response, *n* (%)** | | |  |
| ORR | 0 | 0 | |
| MR | 1 (3.1) | 1 (9.1) | |
| SD | 17 (53.1) | 8 (72.7) | |
| Disease control rate (≥MR or SD ≥8 weeks) | 12 (37.5) | 7 (63.6) | |
| PD | 12 (37.5) | 2 (18.2) | |
| Not evaluable | 2 (6.3) | 0 | |
| **Outcome, months (95% CI)** | | |  |
| Median duration of follow-up | 1.9 (0.8–17) | NA | |
| Median PFS | 1.6 (1–3.2) | NA | |
| Median OS | 10.7 (8–19) | NA | |

*CI* confidence interval, *MR* minimal response, *NA* not applicable, *ORR* overall response rate, *OS* overall survival, *QW/Q2W* once weekly for 4 weeks, once every other week thereafter, *PD* progressive disease, *PFS* progression-free survival, *SD* stable disease.

**Supplementary Table S3. Efficacy of isatuximab based on time from last daratumumab dose to first isatuximab dose.**

| **Best overall response, *n* (%)** | **Time from last daratumumab to first isatuximab** | | | | | |
| --- | --- | --- | --- | --- | --- | --- |
|  | **<3 months**  **(*n*=14)** | **≥3 months**  **(*n*=18)** | **<6 months**  **(*n*=20)** | **≥6 months**  **(*n*=12)** | **<12 months**  **(*n*=27)** | **≥12 months**  **(*n*=5)** |
| MR | 1 (7.1) | 0 | 1 (5) | 0 | 1 (3.7) | 0 |
| SD | 7 (50) | 10 (55.6) | 9 (45) | 8 (66.7) | 14 (51.9) | 3 (60) |
| DCR (≥MR or SD ≥8 weeks) | 4 (28.6) | 8 (44.4) | 5 (25) | 7 (58.3) | 9 (33.3) | 3 (60) |
| PD | 5 (35.7) | 7 (38.9) | 9 (45) | 3 (25) | 10 (37) | 2 (40) |
| Not evaluable | 1 (7.1) | 1 (5.6) | 1 (5) | 1 (8.3) | 2 (7.4) | 0 |

*DCR* disease control rate, *MR* minimal response, *PD* progressive disease, *SD* stable disease.

**Supplementary Table S4. Treatment-emergent adverse events (all-treated population).**

| ***n* (%)** | **Isatuximab 20 mg/kg QW/Q2W**  **(*N*=32)** | |
| --- | --- | --- |
| Patients with any TEAE (any grade) | 28 (87.5%) | |
| Patients with any TEAE of Grade ≥3 | 16 (50.0%) | |
| Patients with any serious TEAE | 13 (40.6%) | |
| Patients with any TEAE leading to death | 4 (12.5%) | |
| Patients with any drug-related TEAE leading to death | 0 | |
| Patients with any TEAE leading to study drug discontinuation | 1 (3.1%) | |
| **TEAEs in at least 10% of the patients by preferred term** | **All grades** | **Grade ≥3** |
| Asthenia | 7 (21.9) | 0 |
| Bronchitis | 6 (18.8) | 0 |
| Infusion reactions | 6 (18.8) | 0 |
| Diarrhea | 5 (15.6) | 1 (3.1) |
| Nausea | 5 (15.6) | 0 |
| Upper respiratory tract infection | 5 (15.6) | 0 |
| Dyspnea | 4 (12.5) | 2 (6.3) |
| Dizziness | 4 (12.5) | 0 |
| Fatigue | 4 (12.5) | 0 |
| Vomiting | 4 (12.5) | 0 |
| Bone pain | 3 (9.4) | 3 (9.4) |
| Cellulitis | 3 (9.4) | 2 (6.3) |
| Back pain | 3 (9.4) | 1 (3.1) |
| Hypokalemia | 3 (9.4) | 1 (3.1) |
| Cough | 3 (9.4) | 0 |
| Decreased appetite | 3 (9.4) | 0 |

*QW/Q2W* once weekly for 4 weeks, once every other week thereafter, *TEAE* treatment-emergent adverse event.

**Supplementary Table S5. Hematological laboratory abnormalities derived from complete blood count, neutrophil count, platelet count and hemoglobin values (all-treated population).**

| **Laboratory abnormality, *n* (%)** | **Isatuximab 20 mg/kg QW/Q2W**  **(*N*=32)** | | |
| --- | --- | --- | --- |
|  | **All grades** | **Grade 3** | **Grade 4** |
| Anemia | 29 (90.6) | 10 (31.3) | 0 |
| Neutropenia | 13 (40.6) | 6 (18.8) | 1 (3.1) |
| Lymphopenia | 22 (68.8) | 9 (28.1) | 1 (12.5) |
| Thrombocytopenia | 23 (71.9) | 1 (3.1) | 6 (18.8) |

*QW/Q2W* once weekly for 4 weeks, once every other week thereafter.


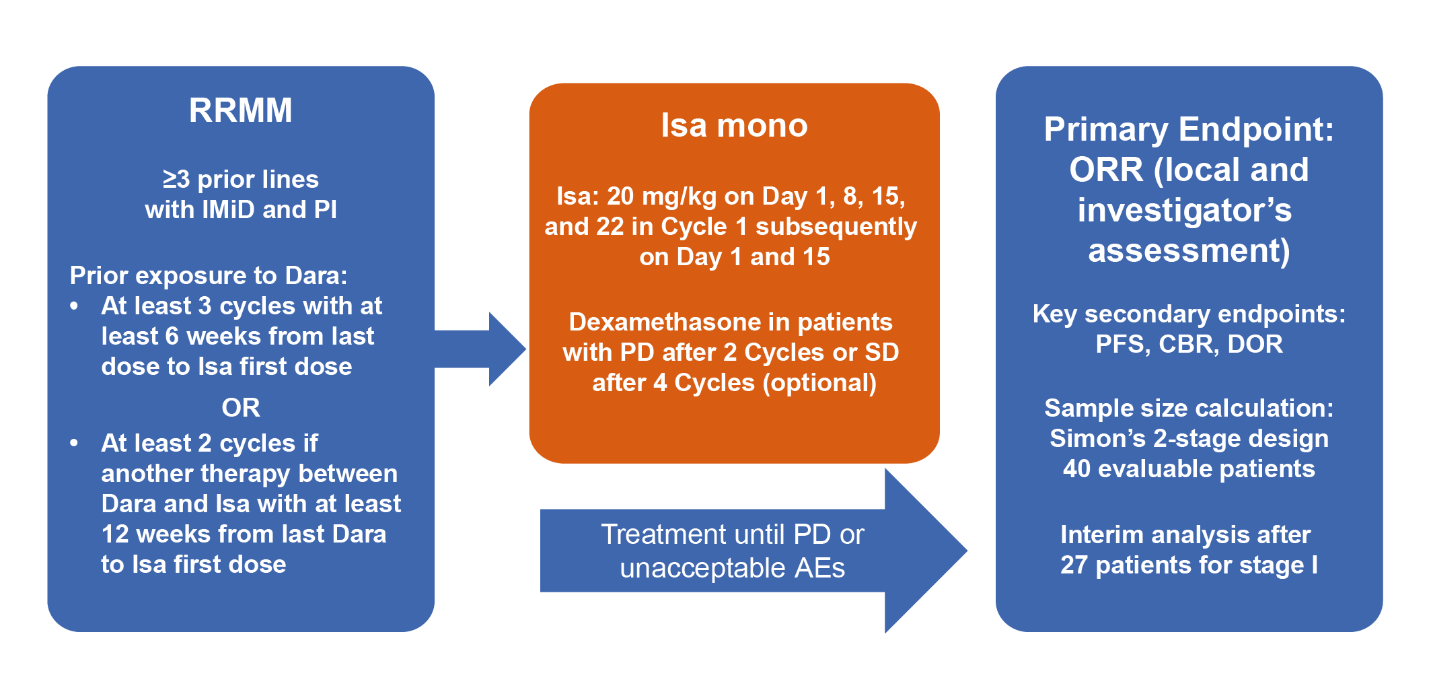


**Supplementary Fig. S1** Study design. Patients with RRMM treated with ≥3 prior lines and previously treated with daratumumab were treated with isatuximab monotherapy. *AE* adverse event, *CBR* clinical benefit rate, *Dara* daratumumab, *DOR* duration of response, *IMiD* immunomodulatory drugs, *Isa* isatuximab, *mono* monotherapy, *ORR* overall response rate, *PD* progressive disease, *PFS* progression-free survival, *PI* proteasome inhibitor, *RRMM* relapsed/refractory multiple myeloma, *SD* stable disease.

**
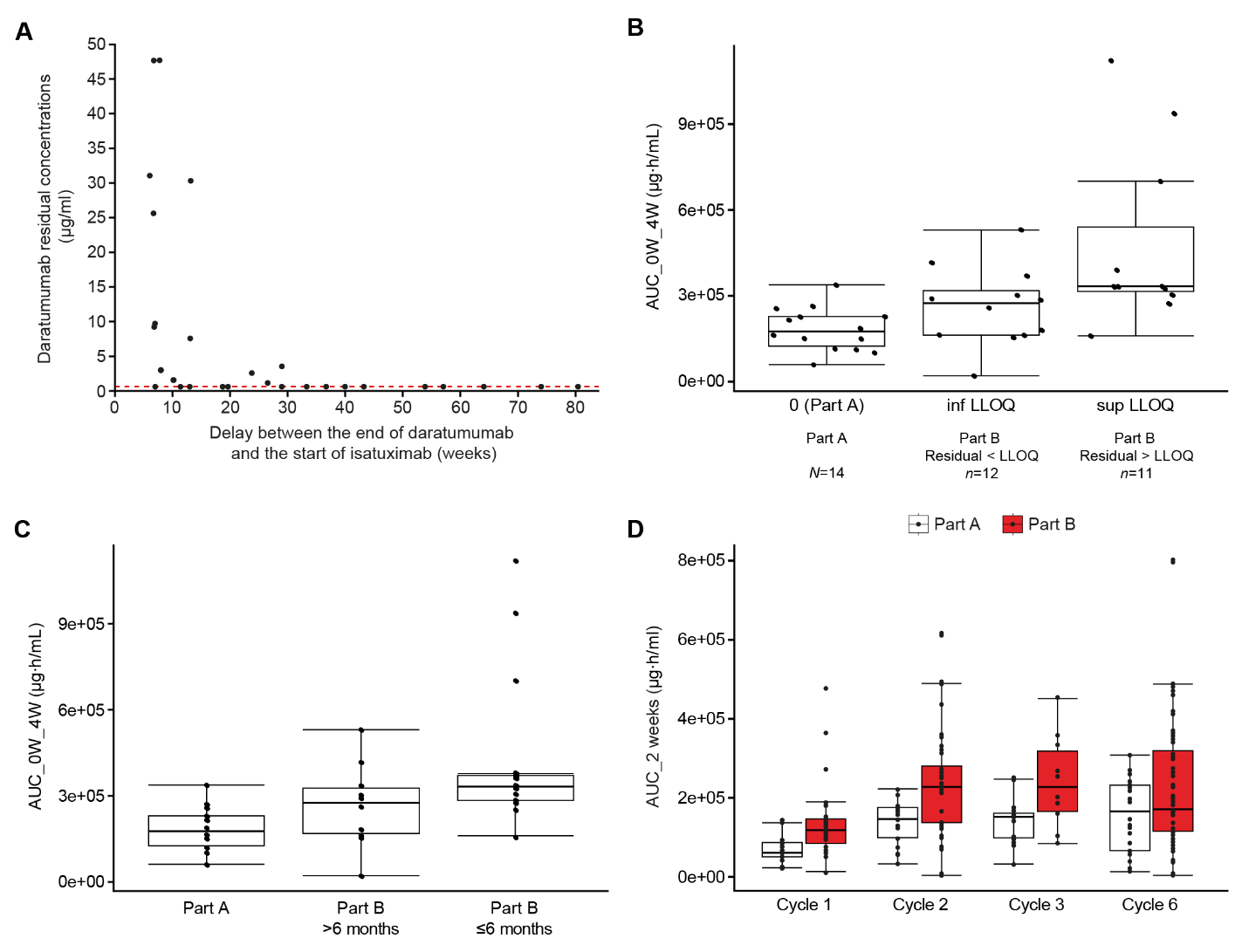
**

**Supplementary Fig. S2** (**A**) Daratumumab residual plasma concentration based on time from last daratumumab dose to first isatuximab dose. (**B**) Boxplot of AUC over the first cycle versus daratumumab residual concentrations (LLOQ = 0.5 µg/mL). (**C**) Boxplot of AUC over the first cycle versus time between the last daratumumab and the start of isatuximab administration. (**D**) Boxplot of predicted isatuximab AUC_2weeks_ at Cycles 1 to 6 in daratumumab-refractory patients (Part B) and daratumumab-naïve patients (Part A) treated with isatuximab 20 mg/kg QW/Q2W. *AUC* area under the curve, *LLOQ* lower limit of quantitation, *QW/Q2W* weekly for 4 weeks and every other week after that.
